# Current Trends for Delirium Screening within the Emergency Department

**DOI:** 10.3390/medicina59091634

**Published:** 2023-09-08

**Authors:** Angela Saviano, Christian Zanza, Yaroslava Longhitano, Veronica Ojetti, Francesco Franceschi, Abdelouahab Bellou, Antonio Voza, Iride Francesca Ceresa, Gabriele Savioli

**Affiliations:** 1 Department of Emergency Medicine, Fondazione Policlinico Universitario A. Gemelli, IRCCS, 00168 Rome, Italy; angela.saviano@policlinicogemelli.it; 2 Department of Anesthesiology and Perioperative Medicine, University of Pittsburgh, Pittsburgh, PA 15260, USA; 3 Italian Society of Prehospital Emergency Medicine (SIS 118), 74121 Taranto, Italy; 4 School of Medicine, Catholic University of the Sacred Heart, 00168 Rome, Italy; 5 Institute of Sciences in Emergency Medicine, Guangdong Provincial People’s Hospital (Guangdong Academy of Medical Sciences), Southern Medical University, Guangzhou 510080, China; 6 Department of Emergency Medicine, School of Medicine, Wayne State University, Detroit, MI 48201, USA; 7 Emergency Department, Humanitas University, Via Rita Levi Montalcini 4, 20089 Milan, Italy; 8 Emergency Room and Internal Medicine, Istituti Clinici di Pavia e Vigevano, Gruppo San Donato, 27029 Milan, Italy; 9 Department of Emergency Medicine, Fondazione Policlinico San Matteo, 27100 Pavia, Italy

**Keywords:** delirium, emergency department, elderly, pain, screening, emergency medicine, sedation, sepsis

## Abstract

Delirium is an acute neurological disorder that involves attention and cognition. It is associated with a high risk of morbidity and mortality among older people (>65 years old). In the context of the Emergency Department (ED), it is frequently experienced by patients but often not recognized. Literature studies have identified some screening instruments for an initial evaluation of delirium. Most of these tools have not been validated yet in the context of emergencies, but, in other settings, they were very useful for assessing and maximizing the recognition of this condition among older patients. We conducted a review of the literature, including randomized control trials, clinical and observational studies, and research studies published in recent years, confirming that most of the screening tools for delirium used in the intensive care unit (ICU) or the geriatric department have not been tested in the ED, and the ideal timing and form of the delirium assessment process for older adults have not been defined yet. The aim of our review is to summarize the updated evidence about the screening tools for delirium in the context of the ED, due to the fact that overcrowding of the ED and the stressful condition of emergency situations (that contribute to the onset of delirium) could expose older patients to a high risk of complications and mortality if delirium is not promptly recognized. In conclusion, we support the evidence that delirium is a current and real condition that emergency physicians have to face daily, and we are aware that more research is needed to explore this field in order to improve the overall outcomes of older patients admitted to the ED.

## 1. Introduction

**Figure 1:**
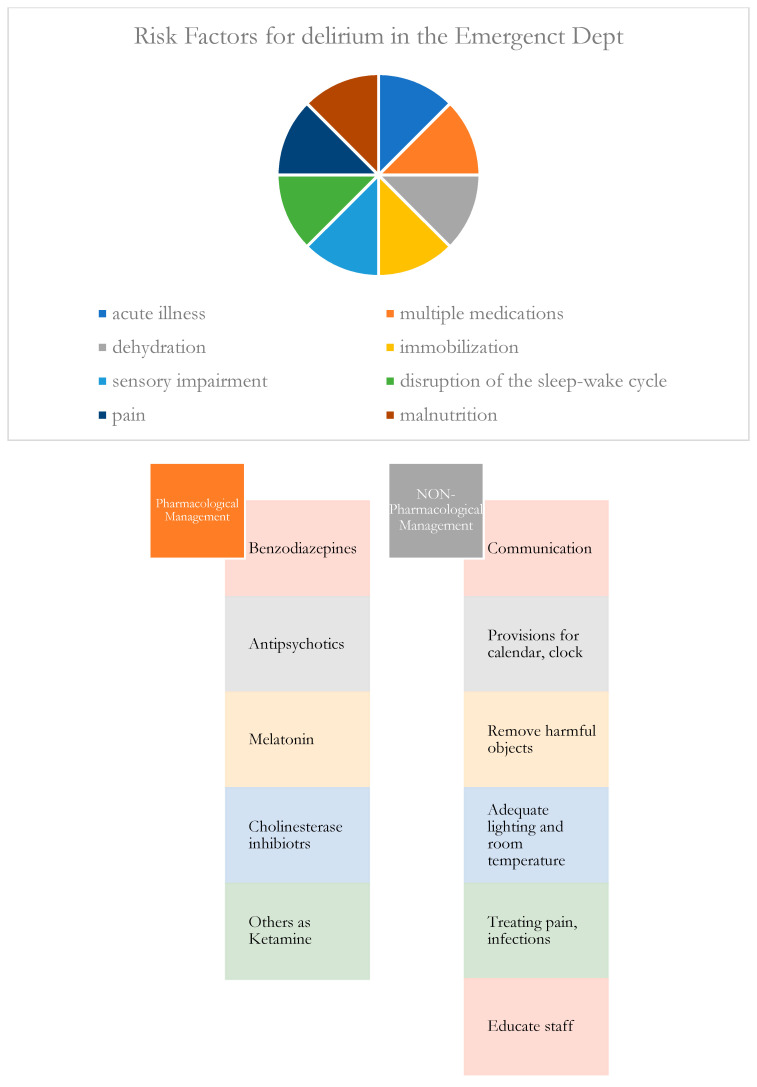
Delirium in the Emergency Department risk factors and management.

Delirium is an acute neurological disorder that involves attention and cognition. It is very common in the context of emergency settings, and it can be very serious and problematic. Literature studies underline that delirium is an acute complex brain dysfunction associated with poor clinical outcomes, longer hospital stays, slower recovery, and frequent readmissions [1]. Often, it is misdiagnosed by emergency physicians (up to 75% of cases estimated [2]) due to several gaps in the screening process, prevention, and management. It is estimated that about 20–30% [3] of older patients (>65 years) admitted to the Emergency Department (ED) presented with delirium. Its identification is fundamental to reducing adverse outcomes and decreasing the high mortality rate associated with this condition [4]. Some research studies have investigated some tools, or quality indicators for cognitive screening upon admission to the ED, with the aim of carefully assessing this disorder. Patients could present confusion, hallucinations, lethargy, restlessness, altered behaviors, alteration in language, memory impairment, impaired level of consciousness, inattention, etc. Comorbidities, advanced age, psychoactive drugs (combined with altered pharmacodynamics and pharmacokinetics due to aging), previous history of alcohol abuse, depression, and previous neurological diseases could contribute to this complex condition (Figure 1). Moreover, infections, sepsis, organ failure, and surgical problems (cardiac, thoracic, orthopedic, abdominal, etc.) can lead to the onset of delirium. There are several tools (Table 1) for detecting delirium, such as 4AT, CAM, inter-RAI-AC, Nu-DESC, PrDICT, Pre-Deliric, RADAR, SQiD, and the Memorial Delirium Assessment Scale, which are not all validated in the emergency setting [1,2,5,6,7,8,9]. Some of them are used as screening instruments, others as diagnostic ones; furthermore, there are also tools to assess the severity of delirium or other tools to evaluate motoric symptoms and cognitive functions. They are considered practical and recommended instruments to assess older patients in order to quickly recognize delirium and prevent its complications, and also have a great potential benefit in the context of an overcrowded ED [7]. 

**Table 1:** Some tools for delirium testing in the Emergency Department.

Screening Tool	Cut-Off Score	Time	Specificity % (95% CI)	Sensitivity % (95% CI)	Setting Validation
The 4 “A”s Test (4AT)	4 or above	<2 min	85%	88–89%	Intensive care unit Emergency setting Acute Medicine Surgery setting
Confusion Assessment Method (CAM)	1, 2, and 3 or 4 items	10 min	85–95%	90–97%	Intensive care unit Emergency setting
Confusion Assessment Method for the Intensive Care Unit (CAM-ICU)	1, 2, and 3 or 4 items	<3 min	88–92%	95–99%	Intensive care unit
Brief Confusion Assessment Method (bCAM)	1, 2, and 3 or 4 items	<2 min	93–99%	80–90%	Critical Care Unit Palliative Care
3-Minute Diagnostic Confusion Assessment Method (3D-CAM)	1, 2, and 3 or 4 items	<3 min	90–98%	85–95%	General medical units Geriatric setting
Delirium Triage Screen (DTS)	2 brief items	<1 min	55–65%	98%	Emergency setting Geriatric/Orthopedics Hematological patients
Spatial Span Forwards (SSF)	<5	<2 min	35–45%	90%	Geriatric setting Neuropsychological Unit
Clock Drawing Test (CDT)	10–15 points scale	<2 min	58–68%	80%	Geriatric setting Memory-Neurological Clinic

## 2. Delirium: Definition, Epidemiology, Risk Factors

Delirium is defined as a confusional state characterized by an acute onset of deficits in attention (truthfully, the Diagnostic and Statistical Manual of the American Psychiatric Association [DSM-5] criteria are broad and applied in many other different acute neurological syndromes) [4,10]. Patients often may experience fluctuation of cognition from agitation and hypervigilance to reduced responsiveness and coma [5]; moreover, they can show disturbances of both attention and awareness and cognitive deficit that can last from a short period to hours or days [5]. Some patients can present changes in speech or mood, alteration of the sleep–wake cycle, disorientation and inattention, and lack of perception [5]. It is a stressful condition for patients and caregivers [11,12]. As regards its reported epidemiologic data from the literature, there is a prevalence of delirium of about 20% among hospitalized patients; >20% in patients undergoing surgery or emergency conditions; 25% after an acute stroke; 35% among patients undergoing palliative care; 50–70% in mechanically ventilated patients; and <2% outside medical institutions [3,6,9,13,14]. In patients with dementia, it can accelerate the severity of this condition [5]. The prevalence of delirium in infants, children, and adolescent populations is not well known (it can range from 4% to 50% in critically ill children) [5]. The wide range of prevalence reflects the numerous factors involved in its pathogenesis (Table 2). A study by Wilson et al. [5] published in *Nature* identifies (a) premorbid risk factors, (b) risk factors related to acute illness, and (c) post-hospitalization risk factors. Premorbid factors include old age (>65 years old), dementia status, frailty, impairment of sight and hearing, low level of education, depression, alcohol or drug/opioid/benzodiazepine abuse, poor nutrition, and a prior history of delirium. Risk factors related to acute illness include sepsis, need for major or minor surgery, dehydration, electrolyte alteration, hypoglycemia, seizures, heart failure, liver or kidney dysfunction, alcohol or drug withdrawal, ventilation, and fractures. Equally important, post-admission risk factors for delirium include pain, infection, blood transfusion, immobility, poor sleep, invasive devices, opioids, benzodiazepines, sedation, day–night disorientation, lack of communication with family, longer duration of ventilation, and placement of physical restraints [8,14]. 

## 3. Delirium: Pathophysiology and Outcomes

Delirium has a complex multifactorial etiology and can be triggered by systemic inflammation, hypoxemia, impaired blood flow, and alteration of electrolytes. The potential biological contributors of delirium have been classified as neurotransmitters, pro-inflammatory markers, physiologic stressors, metabolic disorders, electrolyte disorders, and genetic factors (as apolipoprotein E (ApoE), glucocorticoid receptor, dopamine transporter, and Toll-like receptor 4) [15]. The neurotransmitters most studied include dopamine, acetylcholine, gamma-aminobutyric acid (GABA), melatonin, tryptophan, and serotonin; in addition, glutamate-N-methyl-Daspartate (NMDA) and epinephrine/norepinephrine have been hypothesized to play a role [15]. The identified pro-inflammatory markers include interferon (IFN) α/β, interleukin 6 (IL-6), IL-8, IL-10, tumor necrosis factor (TNF-α), interleukin 1-β (IL 1-β), and prostaglandin E (E2, EP1–4). Interestingly, there are also some physiological stressors such as cortisol, S100B, lactate, hypoxia, or hypercapnia that may activate a cascade of local brain neuroinflammation contributing to delirium [15].

Literature studies have found that cytokines such as interleukin (IL)-1, IL1-beta, IL-6, tumor necrosis factor (TNF), and prostaglandin E2 (PGE2) produced by macrophages and monocytes are able to cross the blood–brain barrier (BBB) and the vascular endothelium up to the brain parenchyma. In addition, these molecules stimulate astrocytes and microglia to produce further pro-inflammatory mediators and chemokines (such as CCL2, CXCL10, and CXCL1) recruiting immune cells and contributing to the amplification of this inflammatory process with subsequent damage of neurons, injury, and cell death [5]. Even if the pathway is not entirely characterized, literature studies found an association with behaviors manifestation of delirium and long-term cognitive decline. In fact, the development of delirium can be the result of four main processes: dysfunction and injury of microglia, increased immune cells infiltration recruited by astrocytes with a consequent unsatisfied metabolic support, vascular dysfunction and neurodegeneration with disturbance of neurotransmitter and reduced integration of brain networks. Inflammation may also contribute to delirium through a state of hypercoagulation that is responsible for cerebral thrombosis and ischemia. Finally, an increased permeability of BBB is under consideration but, to date, no direct evidence exists [5]. Literature studies confirm the multifactorial etiology of delirium. In fact, the development of this condition can be the result of the combination of multiple predisposing risk factors with precipitating factors. In particular, in patients highly vulnerable to developing delirium, for example, those with underlying dementia and multiple comorbidities, a relatively benign “insult “as a single dose of medication for promoting sleep may precipitate up to delirium [14]. Differently, in young patients, delirium may develop after exposure to multiple noxious insults, for example, major surgery, general anesthesia, use of multiple psychoactive drugs, sleep deprivation, and ICU hospitalization. So, the knowledge of this multifactorial etiology of more than a single risk factor may explain why a multifactorial approach can be most effective for both prevention and management of delirium, also in the context of emergency [14,15].

Moreover, studies have been performed on animal models and more research is needed to better elucidate these processes. Outcomes can be very heterogeneous such as the duration of delirium, which can range from a few days up to months. It is estimated that about 20% of patients had persistent delirium (up to six months). The severity of behaviors including attention, memory, and cognition can be different among older patients [2]. The overall outcomes are considered to be poor with an increased incidence of long-term dementia and mortality. For this reason, early recognition of delirium starting from the ED setting can be useful to improve sequels and survival outcomes of patients.

## 4. Screening Tools for Emergency Physicians

### 4.1. 4-A’s Test-4AT

This tool is a 2 min tool often used in general hospitals. It is based on 4 items: 1. alertness (Normal = 0; abnormal = 4); 2. abbreviated mental test-4 (age, DOB, place, year: correct = 0, 1 error = 1, ≥2 errors or untestable = 2); 3. attention, (reaches 7 months = 0, ≥1 error or refuses = 1, untestable = 2); 4. acute change or fluctuation (No = 0, Yes = 4). A result of ≥4 is diagnostic for delirium. Literature studies underline a pooled sensitivity and specificity of 88% [5]. It is easy and quick to use but it may be contraindicated in patients with dementia or other mental impairments [16,17].

### 4.2. Confusion Assessment Method—CAM

It includes four features: 1. acute-onset and fluctuating course in mental status; 2. inattention; 3. altered level of consciousness; and 4. disorganized thinking. It can be used in general hospitals, Intensive Care Units (ICU), palliative units, Emergency Departments and nursing homes. A diagnosis of delirium can be made if features 1 and 2 are present, plus either features 3 or 4. It needs less than 5 min to be completed, even if it can have an additional questionnaire component for a total of 9 diagnostic criteria. Literature studies found a sensitivity and specificity ranging from 94% to 100%, with a NPV of 90–100%. It may be contraindicated in patients with psychiatric disorders. It can have some “variations” based on the setting as CAM-ICU (with a pooled sensitivity of 80% and a specificity of 96%), brief-CAM, pediatric-CAM and preschool-CAM [18,19]. 

### 4.3. The 12-Item Stanford Proxy Test for Delirium—S-PTD

It is a screening tool for non-ICU patients. Nurses can assess the patient for the presence of items suggestive of delirium using 3 or more items of 12 total items. It has a sensitivity and specificity of about 80% [20]. 

### 4.4. Recognizing Acute Delirium as Part of Your Routine—RADAR

It is a tool useful for nurses during the administration of medications. It is based on three questions (Is the patient drowsy? Do they have trouble following instructions? Have the patient’s movements slowed down?) for which an answer of “yes” to one or more items is suggestive of delirium [21]. It is based on observation of patients, there are no direct questions, and it is estimated to take a few seconds (about 7 s) to be completed. It cannot be used if the patient does not have medications to take. Literature studies underline it had a sensitivity of 73% and a specificity of 67% [21]. 

### 4.5. Intensive Care Delirium Screening Checklist—ICDSC

It is composed of eight domains with answers as yes (present; score 1) or no (absent; score 0). Delirium is likely for a score of ≥4 domains [7]. The domains are altered LOC, inattention, disorientation, hallucination, delusions, agitation, inappropriate speech, sleep–wake disturbances, and symptom fluctuation. It has a pooled sensitivity of 74% and a specificity of 82%. It is mainly used in critical or ventilated patients [19].

### 4.6. International Resident Assessment Instrument Acute Care—Inter-RAI-AC

It is composed of four observational items: Acute change in mental status from baseline, mental function that varies over the course of the day, episodes of disorganized speech, and easily distracted. It has a sensitivity of 82% and a specificity of 91%, a PPV of 72% and a NPV of 95% [22]. It has been validated for patients >70 years old. Each item scored from 0 = behavior not present, to 1 = behavior present. It is a useful tool, it acts as a high negative predictor, but it requires a period of 24 h observation [22].

### 4.7. Single Question in DeliriumS—QiD 

The question is “Do you feel that (patient’s name) has been more confused lately?”. Literature studies underline that it can contribute to detect delirium in hospitalized patients. It is a simple and time-efficient screening with a reported sensitivity and specificity of 80% [23]. 

### 4.8. Memorial Delirium Assessment Scale—MDAS

This tool was developed in 1997. It was used to assess the severity of delirium in patients with advanced cancer [24]. It is composed of 10 items rated 0 to 3 points for a maximum total score of 30 points, with higher scores representing more severe delirium. Scores of ≥13 indicate the presence of delirium as revealed in a validation study [25]. It takes ≥10 min to be administered to patients.

### 4.9. Nurse-Based Delirium-Screening Tool—Nu-DESC

The Nu-DESC is an observational screening tool for delirium. It is composed of five items: (1) disorientation, (2) inappropriate behavior, (3) inappropriate communication, (4) hallucination, and (5) psychomotor retardation. Each feature is scored from 0 (absent) to 2 (severe) [26,27,28]. The information has to be collected in a period of 12 h. It takes less than 2 min and it is often used by nursing staff. A score of ≥2 is considered positive for delirium. 

### 4.10. Prediction of Delirium in ICU Patients—PRE-DELIRIC

This tool predicts delirium in the patients within 24 h of admission to the ICU. It has a good specificity and sensitivity of 77% [29]. It is a model that includes ten predictors (age, APACHE-II, urgent and admission category, infection, coma, sedation, use of morphine, level of urea, and metabolic acidosis). Literature studies underline that it may help to prevent delirium and improve the management of ICU patients [29].

### 4.11. Ultra-Brief Screening Tools

These tools are very quick (less than a minute) to administer for detecting delirium [30]. They can be useful for a brief assessment. In case of positivity, they can be completed with a more detailed assessment. For example, the Ultra Brief 2 Item Screener (UB-2) [31,32,33] takes <1 min and consists of two questions: “Please tell me the day of the week?” and “Please tell me the months of the year backwards starting at December?” If either question is not answered correctly, delirium can be suspected and a more definitive tool is required. Another, the Simple Question for Easy Evaluation of Consciousness (SQEEC) [34,35] consists of asking the patient to name a place they would like to visit (not visited before) and then to describe a possible journey. Even if this brief tool has good sensitivity and specificity, they are not appropriate for the regular monitoring of patients [5,31].

### 4.12. Delirium Triage Screen—DTS

Patients have to spell the word ‘lunch’ backward. This tool shows very good sensitivity and specificity; in fact, a negative DTS can be used to rule out delirium. It is very easy and quick to perform [1,3]. 

### 4.13. Spatial Span Forwards—SSF

The SSF is a quick and simple test of attention that can be used in patients with difficulties in language expression. The cut-off of 5 is highly sensitive for delirium; however, it has a specificity of 75% and a sensitivity of 91.7% [36,37]. 

### 4.14. Clock Drawing Test—CDT

The clock drawing test (CDT) is frequently used as a screening instrument for detecting cognitive impairment. It takes <2 min and literature studies reported a good sensitivity of about 80% [38]. It is considered an important additional screening tool for the Mini-mental State Examination (MMSE) [38].

## 5. Discussion

The prevention and early recognition of delirium in the setting of EDs is the most effective strategy to reduce complications in older patients [33,39,40]. Delirium can lead to a decline in the general health of older patients, aspiration pneumonia, decreased mobility, pressure ulcers, weakness, falls, malnutrition, injuries and fractures, electrolyte abnormalities, long-term cognitive impairment, and increased overall mortality [41,42]. Delirium can manifest in two major psychomotor forms: hypoactive delirium and hyperactive [15]. These two forms are clinically different and patients can switch from one form to another during the course of their delirium or a day [15]. In older patients, the predominant form is the hypoactive one, which is often associated with a worse prognosis. For this reason, it is important to early detect delirium manifestations from the admission to the ED. Delirium is often underdiagnosed and undetected in the context of an emergency. Physicians do not always have the ability to detect delirium since they often use alternative diagnoses such as confusion, dementia [43,44], encephalopathy, etc., or they consider this condition to be a normal process of aging.

To evaluate delirium [8,14,15] it is important to collect the patient’s history by family members and caregivers. Emergency physicians need to consider the baseline cognitive function of patients in addition to any recent changes in their mental status. It is important to investigate the new diagnosis received, and recent modifications in the general condition of patients and to review all current home treatments (including also herbal remedies and over-the-counter medications). Emergency doctors should investigate the alcohol habits of patients and the use of psychoactive drugs such as benzodiazepines. A complete assessment of patients with delirium includes also the detection of pain and discomfort (for example, urinary retention with the need for a catheter, constipation, and sensation of thirst), and the observation of vital signs such as oxygen saturation, body temperature, and blood glucose. Neurological and physical examination could help to recognize occult infections, signs of dehydration, pain related to acute abdomen or other acute illnesses such as deep vein thrombosis or fractures, that are risk factors for delirium. Then, the assessment of sensory impairments, focal neurological changes, and/or signs of meningeal infections should be detected. Patients should perform some blood laboratory tests (in particular electrolytes, calcium, sodium, potassium, white blood count cells, and, in selected cases, the dosage of antibodies), tests for liver, kidney, and urinary functions, and thyroid. In the context of an emergency, the dosage of drugs level, the dosage of alcohol and ammonia values, and the toxicology screening are recommended. Others such as vitamin levels could be indicative of the nutritional status of patients. Imaging investigations can include chest X-rays to detect respiratory infections, non-contrast head computed tomography (CT) scans, and head magnetic resonance imaging (MRI) to recognize signs of focal neurological diseases, stroke, and encephalitis. In selected cases, lumbar puncture and electroencephalography are used to evaluate the differential diagnosis of delirium. 

The implementation of educational programs, skills, screening quick tools to early detect and treat delirium can be a challenge for emergency physicians in order to approach older patients and improve their outcomes, from the moment of triage up to hospitalization. Literature studies, as said before, reported an increased length of hospital stay, adverse outcomes, and increased mortality in patients with a diagnosis of delirium [45,46]. To date, guidelines for the emergency setting are not still available and some research is ongoing. Sometimes delirium can manifest subtly together with an underlying serious and potentially life-threatening condition [5,7,8,13,31].

Most conditions underlying delirium are usually reversible if promptly recognized and treated. Guidelines about the management of delirium include the identification of possible etiological factors and the removal/correction of the underlying causes (such as infections, oxygen, and electrolyte imbalance, dehydration, etc.) [47]. Furthermore, to manage symptoms of delirium, pharmacological [48,49] and non-pharmacological treatment [50] can be used [47]. Even if these strategies have been implemented for years both to prevent and treat delirium, their efficacy is still debated until now.

Non-pharmacological treatments include the improvement of communication by using simple words, avoiding abstract language and discussions, reminders of the date, time, and location, face-to-face contact with the patient, providing a calendar and clock, listening to music, a smartphone for relaxation, providing eyeglasses and hearing aids, involving family members/caregivers, protecting the environment by removing harmful objects, using adequate light, reducing noises, encouraging mobilization, ensuring regular bladder and bowel habits, removing unnecessary medications, measures to prevent falls, measures for cognitive and motor rehabilitation, exercises and sleep enhancement [47]. Pharmacological treatments include antipsychotics which are drugs of choice as quetiapine, haloperidol, and chlorpromazine without significant difference between atypical and typical antipsychotics [5,47]; benzodiazepines are indicated mostly in the treatment of alcohol withdrawal and must be initiated low doses due to the risk of excessive sedation; as regards delirium, in clinical practice they are used in hyperactive forms, but some evidence recommended their use with particular care [5,47]. Others are cholinesterase inhibitors such as physostigmine but there are very limited data on highly selective α-2 receptor agonists such as dexmedetomidine with good sedating and anxiolytic properties [47]. Ketamine has shown a role in the treatment of agitation both in the prehospital setting and in hospitalized patients [8,15,47]. But not all emergency staff have knowledge of ketamine protocols that can be used to obtain a safe and effective pharmacological treatment, without significant adverse events. Future studies with standardized protocols are needed to better delineate the role of these drugs in the management of patients with delirium. Today, data are still heterogeneous. Finally, there are few studies that support the use of melatonin to decrease the incidence of delirium in older patients admitted to hospital for its effects on brain activity [5,15]. Most of the evidence seems to agree that there are required “multifactorial interventions” to better manage delirium. In order to perform the best treatment, efforts are necessary to early recognize this condition. Literature reviews offer limited experimental trials and not so much validated scales for delirium in the ED. Differently, some tools have been validated in the context of the ICU or geriatric department.

The ED is a setting of high complexity and workload (even if not all EDs are similar) [51]. Most importantly, the ED setting is often overcrowded (more today than in the past), thus determining an increased risk and rate of adverse events for patients and in particular for the elderly, becoming also specific risk factors for delirium [52,53]. Overcrowding is determined by an imbalance between the healthcare demand that constantly increases, and the lack of availability of hospital beds [54,55,56]. In the context of ED, overcrowding leads to a longer length of stay in the emergency room, a delay in the diagnostic workup of patients and the start of therapy, and, consequently, a greater risk of developing delirium. Interestingly, it has been reported by evidence from the literature that a length of stay of more than 10 h in the ED was associated with a greater risk of episodes of delirium in elderly patients [57]. Another research study confirmed these results, showing that a length of stay of more than 12 h in the ED could lead to episodes of delirium in one older adult out of five patients (results were adjusted for age and cognitive impairment) [58]. Furthermore, overcrowding is responsible for mistakes in the triage process [52,56,59,60], thus negatively interfering with the regulation and coordination of the flow of patients and with the prompt recognition of delirium, worsening the general conditions and outcomes of patients and lengthening the hospital stay as a vicious circle [52,61,62]. Starting from these considerations, it is fundamental to prevent and manage delirium in the ED; as such it is recommended to train emergency physicians and nurses staff about delirium rating scales and the choice of quick screening scales and tools (less than 2–3 min) [43,63]. The instruments such as 4AT, CAM, DTS, UB-2, SQEEC, SQiD score, etc., due to their simplicity and speed of execution (and their good value of sensitivity and specificity) could help to fill the knowledge gap in the assessment of delirium in the older patients admitted to the ED even if more studies are required in this setting. Delirium and research related to this medical condition could have potential implications for public health policies. It can be considered as an indicator of the quality of the health care system [15]. Since the average age of the population is increasing with an intrinsic major risk of developing delirium, efforts must be made mainly in the screening and prevention strategies, without neglecting research on management programs and pharmacotherapy. Improvements may be conducted to achieve an early diagnosis (including a quick idea of severity and subtypes of delirium), educate the medical staff and nurses, and develop cost-effective approaches for the screening (tools for detection, neuroimaging, etc.) and subsequent workup of delirium. Furthermore, literature studies on the long-term follow-up of patients with delirium and an analysis of patients’ experience on genetic predisposition and risk stratification are needed. Health policy should allocate funds for interdisciplinary research, from diagnosis to treatment and follow-up, in different medical settings and also in the context of ED.

## 6. Conclusions

In conclusion, delirium is an acute neurological disorder common among older people (>65 years old) admitted to the ED. Several delirium screening tools to be used in the ED are under investigation. The ideal interval during which the screening process for delirium should take place has not been determined yet. Many EDs are overcrowded and emergency physicians work under pressure so there is an urgent need to have practical and quick tools for the assessments of this condition. The ideal timing and form of the delirium assessment process for older adults has not been identified yet and more studies are required in this setting to better prevent and treat delirium and its complications in older patients and to improve the overall outcomes of patients.

## Figures and Tables

**Table 2:** Risk factors for delirium.

Non-Modifiable Risk Factors	Potentially-Modifiable Risk Factors	Modifiable Risk Factors
Old age		Dehydration
Physical frailty		Electrolytes unbalance
Dementia	Malnutrition	Acute metabolic dysfunction
Genetic predisposition	Sensory and functional impairment	Impaired oxygenation
Disorders of nervous system	Alcohol habits	Acute infections
Previous TIA or stroke	Toxics habits	Post-operative infections
Emergency procedures	Social isolation	Trauma and shock
Cardiac or thoracic surgery		Anemia
Orthopedic procedures		Acute neurological diseases
Multiple medications		Pain
Chronic comorbidities		Catheters
History of falls		Sleep deprivation
Visual, hearing impairment		Emotional distress
Terminal illness		Drugs

## Data Availability

The data that support the findings of this study are available on request from the corresponding author, G.S.
